# The role of study strategy in motivation and academic performance of ethnic minority and majority students: a structural equation model

**DOI:** 10.1007/s10459-018-9840-3

**Published:** 2018-07-25

**Authors:** Ulviye Isik, Janneke Wilschut, Gerda Croiset, Rashmi A. Kusurkar

**Affiliations:** 1 Research in Education, VUmc School of Medical Sciences, P.O. Box 7057, 1007 MB Amsterdam, The Netherlands; 2 LEARN! Research Institute for Learning and Education, Faculty of Psychology and Education, VU University Amsterdam, Amsterdam, The Netherlands; 3 Department of Epidemiology and Biostatistics, VU University Medical Center Amsterdam, Amsterdam, The Netherlands

**Keywords:** Academic performance, Diversity, Ethnicity, Medical students, Motivation, Study strategy

## Abstract

Underperformance among ethnic minority students has been reported in several studies. Autonomous motivation (acting out of true interest or personal endorsement) is associated with better learning and academic performance. This study examined whether study strategy (surface, achieving, and deep) was a mediator between the type of motivation (autonomous and controlled motivation) and academic performance (GPA and clerkship performance), and whether these relations are different for students from different ethnic groups to gain a better understanding about the needed intervention/support in the curriculum. Data was gathered from 947 students at VUmc School of Medical Sciences, Amsterdam. Structural Equation Modelling was performed to test the hypothesized model: a higher autonomous motivation has a positive association with academic performance through deep and achieving strategy, and has a negative association with performance through surface strategy. The model with the outcome variables GPA and clerkship performance had a good fit (n = 618; *df* = 1, RMSEA = 0.000, *p* = 0.43). The model for the ethnic majority and minority groups was significantly different (*p* < 0.025). In this study, autonomous motivation had a positive association with GPA through achieving strategy for the ethnic majority students only. It might be that the size of the minority groups was too small to detect differences or that other factors mediate these relations in ethnic minority students. Qualitative research is needed to identify other factors influencing the academic performance of ethnic minority students and what they experience during their education, in order to support their learning in the right manner.

## Introduction

Underperformance among ethnic minority students in general education, and specifically in knowledge and skills assessments, in medical education has been reported in several studies (Stegers-Jager et al. 2012b; Woolf et al. 2011). Two important factors influencing academic performance are student motivation and study strategy (Kusurkar et al. 2013b). In an earlier study we found differences in the type of motivation of students from different ethnic backgrounds, and also in how it was associated with their GPA (Isik et al. 2017). The role of study strategy in this process and clinical performance grades as an outcome were not explored in this study. The present study aims to explore the relationships between motivation, study strategy, and academic performance for students from different ethnic groups to find out if the interplay of these factors can explain the underperformance.

For the present study we use the framework of Self-determination Theory (SDT) of motivation which classifies types or quality of motivation along a continuum (Kusurkar et al. 2011; Ryan and Deci 2000). Autonomous motivation is the type of motivation which comes out of interest and finding an activity personally important. Controlled motivation comes from internal pressure or external pressure or rewards.

### Motivation and performance of students from the ethnic minorities

In an earlier systematic review exploring factors influencing the motivation of ethnic minority students, we found that 8 out of 11 studies reported higher levels of motivation among minority students (Choi et al. 1994; Gillen-O’Neel et al. 2011; Goodenow and Grady 1993; McInerney 2008; Sentell 2012; Strage 1999; Van Houtte and Stevens 2010; Young et al. 2011), whereas six studies found higher levels of motivation among majority students (Hill et al. 2004; Hill and Wang 2015; Sentell 2012; Strage 1999; Van Houtte and Stevens 2010; Young et al. 2011). The pooled difference between the means of motivation of ethnic minority students with ethnic majority students was considered and no significant difference in motivation was found between the ethnic groups.

In our earlier empirical study, we found significant differences in the type of motivation (autonomous or controlled motivation) between the ethnic groups (Isik et al. 2017). Non-Western minority students showed higher autonomous motivation than Dutch majority students, and Western minority students showed higher controlled motivation than Dutch majority students. Moreover, autonomous motivation was found to positively influence the academic performance of some ethnic groups, like Western students. However, motivation did not directly influence academic performance of non-Western students. The conclusion of this study was that there was no association between the type of motivation and GPA, meaning that other mediating factors could be playing a role.

In addition, previous research reported on the relationship between motivation, study strategy, study effort (the number of hours that students study), and the academic performance of medical students (Kusurkar et al. 2013b). Motivation positively influences the academic performance of medical students through deep study strategy and higher study effort. Considering study strategy could show us whether different ethnic groups use different study strategies which mediate the relation between motivation and academic performance and might explain why no direct relationship was found between motivation and academic performance (GPA) of the different ethnic groups in our previous study (Isik et al. 2017).

### Study strategies

**Fig. 1:**
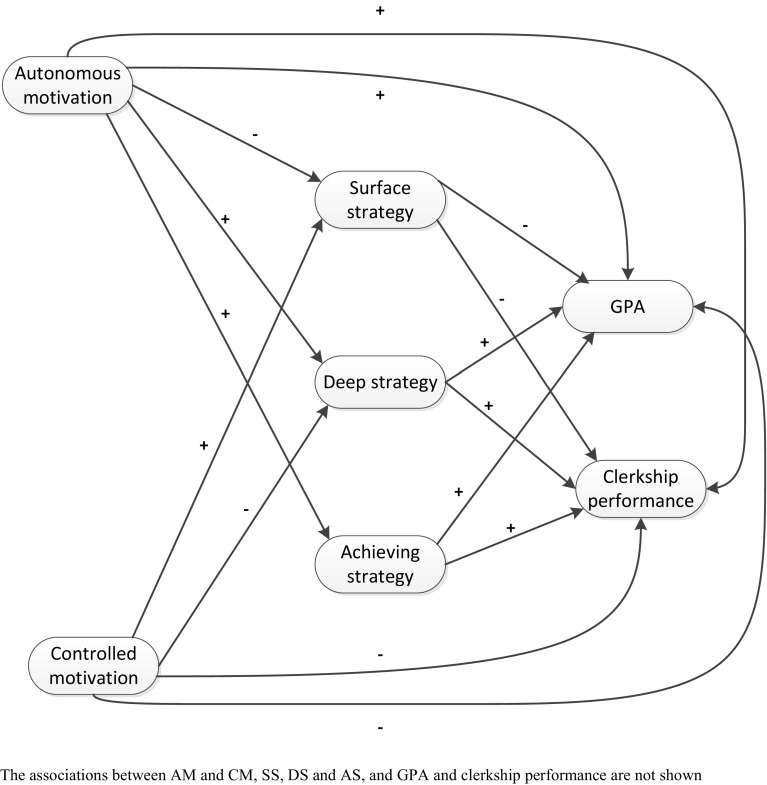
Hypothesized model for motivation influencing GPA and clerkship performance, mediated by study strategy

In the current study, the relationship between the type of motivation (autonomous and controlled motivation), study strategy (surface strategy, deep strategy, and achieving strategy; Biggs 1987), and academic performance (GPA and clerkship performance) for different ethnic groups was investigated (see Fig. 1). Study strategy concerns three types of learning approaches: surface strategy, deep strategy, and achieving strategy. Surface strategy is defined as “rote learning or memorizing the study materials” and is aimed at remembering the facts without understanding their meaning. Deep strategy, the “desired” type, is looking for meaning or to “maximize meaning” in the study materials (Biggs et al. 2001). In general, a positive relationship between deep strategy and learning outcomes and a negative relationship between surface strategy and learning outcomes has been found (Gijbels et al. 2005). Achieving strategy pertains to the effective use of time and space, optimizing efforts, and organizing time and learning strategies to achieve a good grade. The expectation is that students focusing on achieving strategy to earn good grades will perform better than students focusing on surface strategy (Biggs 1987). Academic performance in this study was defined as the performance of students as measured in grades. We investigated study strategy as a mediator in the relationship between motivation (autonomous motivation and controlled motivation) and academic performance and also the differences in the relationships between the ethnic groups. To our knowledge, previous research has not focused on these relationships for medical students from different ethnic backgrounds. The expectation, based on the differences in the type of motivation between ethnic groups (Isik et al. 2017), is that these relationships would be different for the ethnic groups. Moreover, achieving strategy as a mediating variable and the outcome variable clerkship performance have not been investigated before. We included clerkship performance because clinical performance is an equally important part of the medical curriculum as knowledge, and we expect to make specific recommendations for improving clinical performance based on the results.

The aim of this study was to examine the relationships between the type of motivation (autonomous and controlled), study strategy, and academic performance (GPA and clerkship performance) and to determine whether these relationships are different for students from different ethnic groups. Understanding this relationship can inform interventions aimed at helping medical students from different ethnic backgrounds to perform optimally. Based on earlier findings and the literature, our hypotheses for the overall model were (Fig. 1; Kusurkar et al. 2013b; Isik et al. 2017):

1. Autonomous motivation has a positive influence on deep study strategy, which leads to higher GPA and clerkship performance in all medical students.
2. Autonomous motivation has a positive influence on achieving study strategy, which leads to higher GPA and clerkship performance in all medical students.
3. Autonomous motivation has a negative influence on surface study strategy, which leads to a lower GPA and clerkship performance in all medical students.

We also tested this model separately for each ethnic group to explore which path the students from different ethnic groups follow.

## Methods

### Study design and setting

This cross-sectional study was conducted at VUmc School of Medical Sciences, Amsterdam, the Netherlands, as part of a longitudinal study called the “Student Motivation and Success” (SMS) study (Isik et al. 2017). The curriculum at this school has a bachelor-master structure: the bachelor education consists of 3 years of pre-clinical education and is followed by a master phase consisting of 3 years of clinical education (Ten Cate 2007).

### Participants and procedure

All medical students at VUmc School of Medical Sciences were invited to participate in this study through an electronic survey in September 2015 (*n* = 2451). This survey included ethnic background questions, the Academic Self-Regulation Questionnaire (SRQ-A) measuring autonomous and controlled motivation (Vansteenkiste et al. 2005, 2009), and the Study Process Questionnaire (SPQ) measuring surface strategy, deep strategy, and achieving strategy (Biggs 1987). Performance grades were obtained from the school’s student administration. Written informed consent was obtained from the students. Ethical approval was obtained from the Ethical Review Board of the Netherlands Association for Medical Education (NVMO-ERB, dossier number 388).

### Instruments and variables

#### Motivation variables

The scores of autonomous motivation (AM) and controlled motivation (CM) of the participants were measured on a 5-point Likert scale (1 = completely not important, 5 = very important) using a Dutch version of the Academic Self-Regulation Questionnaire (SRQ-A). The average scores on intrinsic motivation and identified regulation together formed AM. The average scores on introjected and external regulation together formed CM. In earlier studies, the Cronbach’s alpha values for reliability varied from 0.63 to 0.88 and from 0.62 to 0.85 for autonomous and controlled motivation subscales, respectively (Vansteenkiste et al. 2009; Wouters et al. 2017a, b).

#### Study strategy variables

The Study Process Questionnaire (SPQ) was used to measure the scores of the variables surface strategy (SS), deep strategy (DS), and achieving strategy (AS) on a 5-point Likert scale (1 = never or only rarely true of me, 5 = always or almost always true of me; Biggs 1987). Surface strategy was measured with items like: “I generally restrict my study to what is specifically set as I think it is unnecessary to do anything extra.” Deep strategy was measured with items like: “In reading new material I often find that I’m continually reminded of material I already know and see the latter in a new light.” Achieving strategy was measured with items like: “I test myself on important topics until I understand them completely” (Biggs 1987).

#### Academic performance variables

Academic performance was measured with the variables GPA and clerkship performance. GPA was measured as the mean of the scores on the first attempt on knowledge tests that the participating students had taken so far and weighted by relative score of all respondents compared to the average score of all students in the same study phase (on a scale of 1–10; 1 = poor and 10 = excellent). Clerkship performance (including clerkship grades of different years of medical study) was operationalized as grades from 1 to 10 (1 = poor and 10 = excellent) and measured with the weighted mean of the available scores. Academic performance rates were retrieved from the student administration database.

#### Ethnic background questions

Questions about the ethnic background of the students pertaining to the country of birth of the student and parents and the language spoken with parents were included in the survey (Stegers-Jager et al. 2012b). Ethnic minority was defined according to the Statistics Bureau of the Netherlands (CBS, www.cbs.nl) as “a person with at least one parent born outside the Netherlands.” The classification of ethnic minorities in five groups was also in alignment with CBS: “Turkish/Moroccan/African, Surinamese/Antillean, Asian (including Chinese), Western (including European, North American and Oceanian, Indonesian, and Japanese), and Other” (Stegers-Jager et al. 2012b). The size of some ethnic groups were too small, so we decided to combine them and made three final ethnic groups: Dutch (majority group), Western (minority group including European, North American and Oceanian, Indonesian, and Japanese), and non-Western (minority group including Turkish/Moroccan/African, Surinamese/Antillean, Asian and Other).

### Statistical analysis

Data were checked for missing values and normality distribution. Reliability tests were performed for the scales used in the study. Pearson’s correlations of all variables were computed. Comparison of scores on all variables between the ethnic groups was performed using univariate analysis of variance (ANOVA). A Bonferroni correction was used in the post hoc analysis to account for multiple comparisons. These analyses were performed using the SPSS 22.0 software program.

Structural equation modelling (SEM) is a confirmatory approach that evaluates multiple hypothesized relationships between variables (Bollen and Pearl 2013; Violato and Hecker 2007). SEM analysis was performed to explore the relationships between motivation (autonomous motivation and controlled motivation), study strategy as a mediator, and academic performance for the different ethnic groups. SEM was performed using the Mplus 7.0 software program. The recommended sample size for SEM is a minimum of 200 cases (Kline 2016). The indicators that were used as a good fit of the SEM models were: Chi Square test of model fit > 0.05, root mean square error of approximation (RMSEA) < 0.05, and comparison of fit index (CFI) > 0.9 (Hair et al. 2010; Kline 2016). Multi group analysis was performed to assess the differences in the relations between the sub-groups. Subsequently, the estimates between the ethnic groups were tested for similarity using a Wald test.

## Results

### Student characteristics

The response rate of the SMS study was 38.6% (947 out of 2451 students). Some students were excluded because their reported student number was not registered (n = 72), and one student could not be categorized into a single ethnic group. Since the objective of the study was to look for similarities and differences between ethnic groups, we left out the students who did not report their ethnic background and could not be considered within one of the ethnic groups (n = 217) from all analysis. Finally, another 39 students were omitted from the analysis because they did not fill in any of the questions of one of the constructs used. The ethnicity of the students omitted did not differ from the whole population. 618 students were included in the analyses. The gender distribution was representative for the Dutch medical student population, 24.4% male (n = 151) and 75.6% female (n = 467; Ten Cate 2007). The categorization of the students was as follows: 77.2% Dutch majority students (n = 477), 8.4% Western minority students (n = 52), and 14.4% non-Western minority students (n = 89).

### Preliminary analyses

#### Reliabilities and correlations

The Cronbach’s alpha for reliabilities of the used scales were autonomous motivation = 0.85, controlled motivation = 0.85, deep strategy = 0.72, surface strategy = 0.57, and achieving strategy = 0.76, which were comparable with other studies (Biggs et al. 2001; Kusurkar et al. 2013b). Correlational analysis of the variables was conducted to check the relationships in the hypothesized model (Fig. 1). Autonomous motivation was significantly positively correlated with surface strategy, deep strategy, achieving strategy, and GPA and significantly negatively correlated with controlled motivation (Table 1). Controlled motivation was significantly negatively correlated with achieving strategy. Surface strategy was significantly positively correlated with achieving strategy and significantly negatively correlated with deep strategy. Deep strategy was significantly positively correlated with achieving strategy and GPA. Achieving strategy was significantly positively correlated with GPA. GPA was significantly positively correlated with clerkship performance.

**Table 1:** Pearson’s correlations between the variables (N = 618)

Variables	AM	CM	SS	DS	AS	GPA	Clerkship performance
AM	–						
CM	− 0.24**	–					
SS	0.08*	0.05	–				
DS	0.39**	− 0.04	− 0.14**	–			
AS	0.40**	− 0.12**	0.17**	0.38**	–		
GPA	0.13**	− 0.05	− 0.05	0.16**	0.28**	–	
Clerkship performance	0.03	0.02	− 0.05	0.05	0.03	0.24**	–

*SE* standard error, *AM* autonomous motivation, *CM* controlled motivation, *SS* study strategy, *DS* deep strategy, *AS* achieving strategy, *GPA* grade point average

*p < 0.05; **p < 0.01

#### Comparison of the variables between ethnic groups

We compared the scores of all variables between the ethnic groups using univariate ANOVA. The variables autonomous motivation and deep strategy were significantly different between the ethnic groups (Table 2). The autonomous motivation of non-Western students was significantly higher than that of Dutch students (*p* < 0.001). The deep strategy of non-Western students was significantly higher than that of Dutch students. There were no significant differences in controlled motivation, surface strategy, achieving strategy, clerkship performance, and GPA between the ethnic groups.

**Table 2:** Results of univariate ANOVA comparing the ethnic groups (N = 618)

Variables	Dutch majority (N = 477) - Mean (SD)	Western minority (N = 52) - Mean (SD)	Non-western minority (N = 89) - Mean (SD)
AM	4.25* (0.50)	4.29 (0.55)	4.47* (0.46)
CM	1.83 (0.65)	2.03 (0.78)	1.99 (0.69)
SS	2.95 (0.57)	2.91 (0.47)	3.03 (0.55)
DS	3.18* (0.60)	3.24 (0.67)	3.35* (0.64)
AS	3.12 (0.85)	2.97 (0.79)	3.05 (0.86)
GPA	6.78 (0.78)	6.89 (0.93)	6.73 (0.83)
Clerkship performance	7.56 (0.82)	7.37 (1.06)	7.58 (0.78)

*Significantly different between Dutch and non-Western; *p* < 0.05

### Structural equation model

#### Hypothesized model for all students

**Fig. 2:**
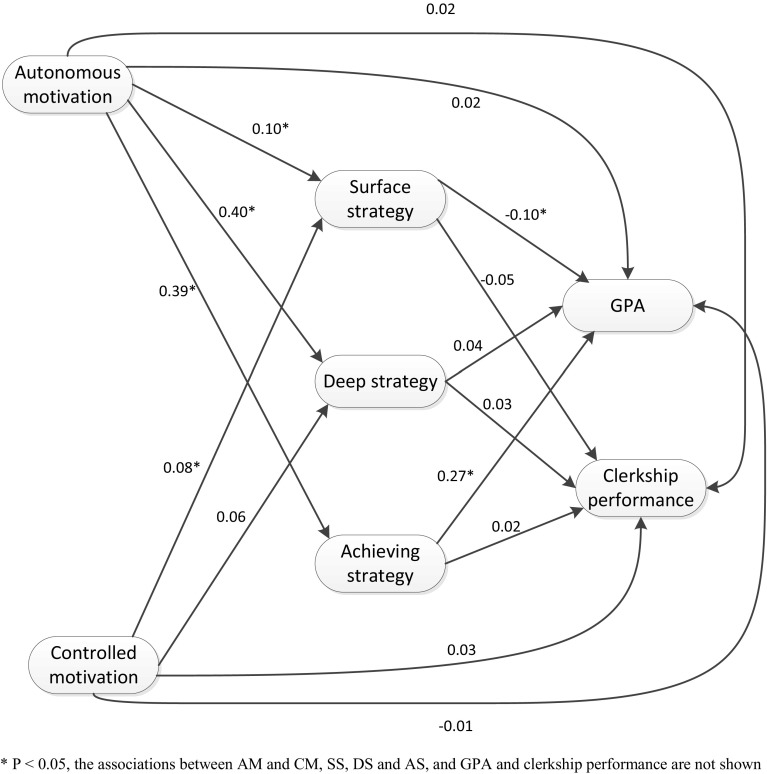
Final model SEM (with standardised regression coefficients)

The structural equation model analyses with the outcome variable academic performance (GPA and clerkship performance) resulted in the model pictured in Fig. 2 (n = 618); this model had a good fit (*df* = 1, Chi square = 0.64, *p* = 0.43, RMSEA = 0.000 (< 0.05), and CFI = 1.000, > 0.9). As Fig. 2 shows, autonomous motivation was positively associated with achieving strategy, which was in turn, positively associated with GPA (Hypothesis 2). Autonomous motivation was not directly related to GPA, but only via achieving strategy (β = 0.11, SE = 0.02, *p* < 0.001). In addition, autonomous motivation was positively associated with surface strategy, which was in turn, negatively associated with GPA (Hypothesis 3). The total effect of autonomous on GPA via surface strategy however was non-significant. Autonomous motivation was also positively associated with deep strategy. Controlled motivation was positively associated with surface strategy. The other associations were not significant.

#### Hypothesized model compared between ethnic groups

The multi-group analysis to test for differences between the three ethnic groups showed a significant difference between the groups (Δ *df* = 34, Δ Chi square = 59.1, *p* < 0.025). The model with parameters freely estimated for the ethnic groups was good. (*df* = 3, Chi square = 2.58 *p* = 0.46, RMSEA = 0.000 (< 0.05), and CFI = 1.000, > 0.9). The group-specific estimates showed in which subgroups the relationships were confirmed by statistical significance (Table 3). Autonomous motivation was significantly positively associated with the use of surface strategy, deep strategy, and achieving strategy by all three ethnic groups except autonomous motivation and the use of surface strategy by Western students, which was negatively associated. Further, the use of achieving strategy was significantly positively associated with GPA only for Dutch students. Autonomous motivation was related to GPA via achieving strategy only for Dutch students (β = 0.14, SE = 0.03, *p* < 0.001). Controlled motivation was significantly positively associated with the use of surface strategy only for non-Western students. The other associations were not significant. The relations that differed significantly between the ethnic groups were the relations between autonomous motivation and surface strategy and between autonomous motivation and deep strategy, and the relation between surface strategy and clerkship performance.

**Table 3:** (Standardised) regression coefficients and standard error of variables between SEM models for Dutch students, Western students, and non-Western students

	Dutch majority β (SE)	Western minority β (SE)	Non-Western minority β (SE)
AM on SS^†^	0.10* (0.05)	− 0.29* (0.13)	0.22* (0.10)
AM on DS^†^	0.38*** (0.04)	0.66*** (0.08)	0.33*** (0.10)
AM on AS	0.43*** (0.04)	0.37** (0.12)	0.25* (0.10)
AM on GPA	0.01 (0.05)	0.26 (0.17)	− 0.19 (0.10)
AM on clerkship performance	0.05 (0.05)	0.09 (0.18)	− 0.22 (0.11)
CM on SS	0.05 (0.05)	0.05 (0.13)	0.22* (0.10)
CM on DS	0.08 (0.04)	0.03 (0.10)	− 0.09 (0.09)
CM on GPA	− 0.01 (0.05)	− 0.01 (0.13)	0.03 (0.11)
CM on clerkship performance	0.05 (0.05)	− 0.05 (0.13)	0.02 (0.11)
SS on GPA	− 0.01 (0.05)	− 0.18 (0.14)	− 0.08 (0.12)
DS on GPA	0.02 (0.05)	0.06 (0.18)	0.13 (0.13)
AS on GPA	0.33*** (0.05)	0.05 (0.15)	0.12 (0.28)
SS on clerkship performance^†^	− 0.06 (0.05)	− 0.27 (0.14)	0.20 (0.12)
DS on clerkship performance	0.02 (0.05)	− 0.14 (0.19)	0.24 (0.13)
AS on clerkship performance	0.01 (0.05)	0.15 (0.16)	− 0.04 (0.12)

*SE* standard error, *p < 0.05; **p < 0.01; ***p < 0.001, ^†^significantly different between the ethnic groups

## Discussion

This study set out to examine the relationships between the type of motivation (autonomous motivation and controlled motivation), study strategy, and academic performance (GPA and clerkship performance) and whether these relationships are different for students from different ethnic groups. Our study adds to the existing literature because earlier research has not included achieving strategy and clerkship performance in their models (Kusurkar et al. 2013b).

### Findings for all students

Autonomous motivation was positively associated with surface, achieving, and deep strategy in all students. Not all associations were in turn related to academic performance. Based on earlier studies the positive association between surface strategy and autonomous motivation was not expected (Kusurkar et al. 2013b; Sobral 2004). Probably, the types of questions asked in the assessments used in our medical curriculum drive this type of perceptions and behaviour among students.

Contrary to our Hypothesis 1, and earlier research (Kusurkar et al. 2013b), we did not find deep strategy as a mediator between the variables autonomous motivation and academic performance (Hypothesis 1). The SEM models in some other studies have also showed that deep strategy was not related to the academic performance of medical students (Heijne-Penninga et al. 2010; Stegers-Jager et al. 2012a). However, as expected in Hypothesis 2, the findings showed that autonomous motivation is positively associated with the use of achieving strategy by students, which is in turn, positively associated with higher GPA (Hypothesis 2). Further, contrary to our Hypothesis 3 and earlier research, we found that autonomous motivation is positively associated with surface strategy by students, which is in turn, associated with lower GPA. Controlled motivation was positively associated with surface strategy, which was in turn, negatively associated with GPA. This was expected because controlled motivation and surface strategy have been reported to be associated with negative academic performance (Gijbels et al. 2005; Kusurkar et al. 2013a; Vansteenkiste et al. 2009). However, the indirect effects for these variables were not significant in the present study. An explanation for these findings could be that the students use more achieving strategy than deep strategy because this strategy is more efficient to pass their knowledge tests. It might also be that the medical curriculum drives students to use surface and achieving strategy because of the types of assessments used or the ways in which learning goals are formulated. Another part of Hypothesis 2 was a positive association between autonomous motivation and clerkship performance, with achieving strategy as a mediator; however, these associations were not found. These findings indicate that other factors might play a role in the motivation and clerkship performance of the students; for example, clerkship performance could be associated with teaching behaviour (Roop and Pangaro 2001). Roop and Pangaro (2001) found that the teachers’ educational skills (as rated by students) had a positive influence on clerkship performance.

The findings showed that autonomous motivation, controlled motivation, and study strategy were not associated with clinical performance. We used the Academic Self-Regulation Questionnaire to measure autonomous and controlled motivation for studying medicine in general (Stem of the questionnaire was “Why do you study medicine?”) and not specifically for clinical skills/performance. The Study Process Questionnaire (measuring study strategy) does not measure study strategy in a clinical setting. This could explain why the variables autonomous motivation, controlled motivation, and study strategy were not associated with clerkship performance.

### Comparison between ethnic groups

Achieving strategy was a mediator only between autonomous motivation and GPA for Dutch majority students. Autonomous motivation was positively related to surface, deep, and achieving strategy in all groups except for the Western group, which showed a negative association between autonomous motivation and surface strategy. Further, controlled motivation was significantly positively associated with the use of surface strategy only for non-Western students. Other studies have also showed that controlled motivation is associated with the use of surface strategy (Kusurkar et al. 2013a, b; Sobral 2004). In addition, one of our earlier studies has reported that Western students were more controlled motivated than Dutch students and more often had a doctor as a parent than Dutch and non-Western students. This indicates that Western students may feel more internal and external pressure to become a doctor like their parents (Isik et al. 2017). The other associations were not significant.

We found differences in the relationship between autonomous motivation and study strategy for Dutch and non-Western students. Dutch students focused more often on using achieving strategy to get high grades and non-Western students focused on using deep strategy more often than achieving strategy. In a situation, where knowledge assessments are not geared to test deep knowledge and are not sensitive enough to distinguish between the output (i.e., GPA), the lack of significant differences is not surprising. In this study and our earlier study, we have shown that non-Western students were more autonomously motivated than Dutch students (Isik et al. 2017), and autonomous motivation was positively related to deep learning strategy in another study (Kusurkar et al. 2013a, b); this could be the case for non-Western students.

### Limitations

A limitation of this study is the low response rate (38.6%). This was in particular the case for the ethnic minority groups (14.3% non-Western, 8.4% Western) compared to the Dutch majority group (77.3%). At the beginning of the study year, September 2015, 69.5% Dutch majority students, 20.9% non-Western students, and 9.6% Western students were enrolled as medical students. It seems that the participants with an ethnic minority background are not representative for all ethnic minority medical students at the school.

Another limitation is the low Cronbach’s alpha of the subscale surface strategy (SS = 0.57). Because of the large sample size (n = 873), we decided to use this subscale. The Cronbach’s alpha of surface strategy was also in line with other studies (Biggs et al. 2001; Kusurkar et al. 2013a, b). SEM is based on theoretically-based hypotheses, but it is not possible to infer causality on the basis of the found model. It only shows that the causal assumptions are more plausible (Bollen and Pearl 2013). The use of self-report measures was also a limitation of this study, and it may have led to social desirability bias.

### Implications

Autonomous motivation, controlled motivation, and study strategy were not associated with clinical performance in the present study. The Academic Self-Regulation Questionnaire measured autonomous and controlled motivation for studying medicine in general and not specifically for clinical skills/performance. The Study Process Questionnaire also measured mainly approach towards studying for building medical knowledge and not clinical practice which involves a combination of knowledge and skills. This might be the reason why the variables autonomous motivation, controlled motivation, and study strategy were not associated with clerkship performance in our study. We conclude that the relation between the type of motivation and clerkship performance cannot be investigated using a questionnaire that measures motivation for studying medicine in general. We recommend using a questionnaire measuring motivation specifically for clinical practice. We also conclude that the Study Process Questionnaire can be used only for investigation of approach toward studying medical content/cognitive knowledge and not for investigating learning approaches used in clinical practice.

Furthermore, this study made it clear that achieving strategy mediates the relationship between autonomous motivation and GPA for all students (Fig. 2). In all likelihood, the medical curriculum leads the students to use achieving strategy because of the forms of assessments used or the ways in which learning goals are formulated. Based on these findings, we recommend that medical schools choose assessments methods or learning goals that motivate the use of deep strategy among students. Also, as far as we know this is the first study investigating achieving strategy in medical students and this study brought to light the importance of using achieving strategy in order to achieve higher GPA in medical students. Based on this finding we recommend that achieving strategy should be investigated in further studies in medical students to find out why this strategy does not lead to higher GPA in ethnic minority students. Furthermore, Non-Western minority students are more autonomously motivated and use more the “desired” type of study strategy than the Dutch majority students, however this in turn does not lead to a higher GPA. Based on these findings we recommend that qualitative research is needed to search out this finding and to identify other factors influencing academic performance, especially among ethnic minority students and what these students experience during their education to acquire a better understanding about how to support their learning.

## Conclusion

The following conclusions could be made based on our findings:

- Students, even if they are more autonomously motivated for medicine, have an increased score on all three study strategies for learning. However, not all associations were in turn related to academic performance (Hypotheses 1–3).
- Higher autonomously motivated students are more likely to use achieving strategy, which results in higher GPA. This was not the case for ethnic minority students (Hypothesis 2). The Dutch students seem to use achieving strategy more effectively than the other groups.
- Autonomous motivation, controlled motivation, and study strategy are not associated with clinical performance. We conclude that the relation between the type of motivation and clerkship performance cannot be investigated using a questionnaire that measures motivation for studying medicine in general. We recommend using a questionnaire measuring motivation specifically for clinical practice.
- An in-depth exploration of these relationships using qualitative research is needed. Also qualitative investigation of other factors influencing ethnic minority students’ motivation and academic performance is needed to be able to design interventions aimed at helping these students to perform optimally.
